# Elderly HIV-positive women: A gender-based analysis from the Multicenter Italian “GEPPO” Cohort

**DOI:** 10.1371/journal.pone.0222225

**Published:** 2019-10-17

**Authors:** Emanuele Focà, Paola Magro, Giovanni Guaraldi, Agostino Riva, Anna Maria Cattelan, Giuseppe Vittorio De Socio, Cecilia Costa, Stefania Piconi, Benedetto Maurizio Celesia, Silvia Nozza, Giancarlo Orofino, Antonella Castagna, Giovanni Di Perri, Francesco Castelli, Andrea Calcagno, on behalf of the GEPPO (GEriatric Patients living with HIV/AIDS: a Prospective Multidimensional cOhort) Study Group

**Affiliations:** 1 Division of Infectious and Tropical Diseases, Department of Clinical and Experimental Sciences, University of Brescia, Brescia, Italy; 2 Infectious Diseases Clinic, Department of Mother, Child and Adult Medicine and Surgical Science, University of Modena and Reggio Emilia, Modena, Italy; 3 Third Division of Infectious Diseases, University of Milan, Ospedale L. Sacco, Milano, Italy; 4 Unit of Infectious Diseases, Department of Internal Medicine, Azienda Ospedaliera-Universitaria di Padova, Padova, Italy; 5 Department of Infectious Diseases, Azienda Ospedaliero-Universitaria di Perugia, Perugia, Italy; 6 Unit of Infectious Diseases, Department of Medical Sciences, University of Turin, Turin, Italy; 7 First Division of Infectious Diseases Unit, University of Milan, Ospedale L. Sacco, Milan, Italy; 8 Division of Infectious Diseases, University of Catania, ARNAS Garibaldi, Catania, Italy; 9 Department of Infectious Diseases, San Raffaele Scientific Institute, Milan, Italy; 10 Unit of Infectious Diseases, Division A, Ospedale Amedeo di Savoia, Turin, Italy; Yeshiva University Albert Einstein College of Medicine, UNITED STATES

## Abstract

**Background:**

HIV-positive patients are facing age-and disease-related comorbidities. Since gender differences in viro-immunological, clinical and therapeutic features have been described, aim of this analysis was to explore such differences in elderly HIV-positive females compared to males coming from the same cohort.

**Design:**

Cross-sectional study.

**Setting:**

Ten Infectious Diseases Center participating to a new multicenter Italian geriatric Cohort aiming at describing health transition over time in HIV-positive individuals.

**Participants:**

HIV-positive patients aged ≥65 years old.

**Measurements:**

We recorded clinical, viro-immunological and therapeutical data.

**Results:**

We included 210 women (17%) out of 1237 patients. Compared to males, elderly females were less likely to present a HIV-RNA <50 copies/mL (74.3% vs. 81.8%, OR 0.64, 95%CI 0.44–0.93); they showed higher CD4+/CD8+ ratio (p = 0.016). Combined antiretroviral therapy (cART) strategies were similar between genders (p>0.05), although women were less likely to be treated with protease Inhibitors (PIs) (p = 0.05); specifically, in triple-drug regimens females received less PIs (28% vs 38% p = 0.022) and more integrase inhibitors (30% vs. 20% p = 0.012). Bone disease was more common in females (p<0.001) while males presented more frequently cardiovascular disease (CVD) (p<0.001). In females with bone disease, PIs and boosted regimens (38% vs. 53.7% p = 0.026 and 30.4 vs 44.0% p = 0.048 respectively) were prescribed less frequently. Polypharmacy was common and similar in both genders (20% vs. 22.8%, p = >0.05). A higher use of lipid-lowering drugs (20.5% vs. 14.8%, p = 0.04) was observed in females and yet they were less likely to receive anti-thrombotic agents (18.6% vs. 26.3%, p = 0.019) even when CVD was recorded (57.1% vs. 83.1%, p = 0.018). **In multivariate analysis, we found that female gender was independently associated with a higher CD4+/CD8+ ratio but not with virological suppression**

**Conclusions:**

Elderly HIV-positive women display a worse virologic response despite a better immune reconstitution compared to males. The burden of comorbidities as well as the medications received (including cART) may slightly differ according to gender. Our data suggest that more efforts and focused interventions are needed in this population.

## Introduction

HIV epidemic has been changing dramatically in the last decades and nowadays a growing number of people living with HIV are aged 50 years and over. According to the last estimates, this number reaches 3.6 million people worldwide [1]. In fact, thanks to combined antiretroviral therapy (cART), HIV-positive patients experienced a reduction of mortality and a consequent increase in life expectancy, which nowadays is similar to that of the general population [2]. Moreover, due to low perception of the risk of HIV acquisition and lower awareness of HIV disease, more people are contracting HIV in their middle and older ages [3]. Sexually active post-menopausal women may be less likely to use condom as long as they are not worried about becoming pregnant [4]. Moreover, the physiological thinning and increased dryness of the vaginal mucosa after menopause may expose elderly women to a higher risk of HIV transmission [5]. Smit *et al* estimated that by 2030, 73% of HIV infected patients will be aged 50 years or more. Therefore, these patients will be facing HIV-related together with age-related comorbidities [6–8], as well as the presence of several medications to treat them [9].

Even if still on debate [10,11], it has been generally accepted that geriatric age starts after 65 years old. Only few cohort studies assessed the clinical features of elderly HIV-positive persons, where most of them did not focus their studies on geriatric age defined as ≥65 years old and included younger patients in their studies [12–14]. To date, 51% of people living with HIV globally are women [15] and older women accounted for the 23% of new HIV diagnosis among people aged 50 years or older in the United States in 2014 [4]. In a recent study from Allavena *et al*, females represented around the 25% of the aging population of the French Dat’AIDS cohort [16]. However, females keep on being less represented than males in clinical studies [17], where very little is known about specific characteristics of elderly HIV-positive women. Some differences in HIV infection between genders have already been pointed out in some studies with regard to the risk of HIV acquisition [18], viro-immunological response [19–22] and treatment choices [23], where some of these seems to be driven by socio-economic and cultural differences between men and women, rather than by different responses to the virus itself. Therefore, aim of this study is to explore the characteristics of elderly HIV infected women from either a viro-immunological, clinical and therapeutic point of view in comparison to their male counterparts, in order to better characterize this population and its special needs for a better management of these patients in the near future.

## Methods

We performed a cross-sectional, retrospective study, including HIV-positive patients aged ≥65 years old. We retrieved data from the GEriatric Patients living with HIV/AIDS cOhort (GEPPO). This is a prospective, observational, multicenter cohort including HIV-positive geriatric patients in follow-up by 10 HIV clinics in Italy. Main cohort inclusion criteria are: age ≥65 years, confirmed HIV-positivity, being on cART for at least 6 months. Aim of this cohort is to describe health status and transition over time in HIV-positive patients aged 65 years and older, in order to better understand the process of aging with HIV infection. In this study, we wanted to explore the existence of viro-immunological, clinical and/or therapeutic differences between the female and male population. Therefore, we recorded clinical, viro-immunological and therapeutic data at initial visit, which was performed between June 2015 and May 2016. The following data were retrieved: age, ethnicity, HIV duration, CD4+ and CD8+ cells count, CD4+ nadir, HIVRNA, HCVAb, HBsAg, actual cART regimen and co-medications. Moreover, we explored the presence of the following comorbidities: cardiovascular disease (CVD), chronic kidney disease, hypertension, type 2 diabetes mellitus, bone disease, hyperlipidemia, chronic obstructive pulmonary disease and cancer, where each condition has been previously described in a more extensive way [24]. We defined polypharmacy as the prescription of ≥5 drugs not taking into account cART.

Non-parametric tests were used for all analysis. Mann-Whitney test was used for comparing continuous variables among female and male participants. Categorical variables were compared through Chi-square and Fisher exact tests (the latter when less than 5 participants fell into one of the categories of the contingency tables). Two step-wise multivariate analysis were used for estimating independent predictors of virological suppression (binary logistic regression) and CD4/CD8 ratio (linear logistic regression) including variables that showed significant p-values at bivariate analysis. All analysis were performed through SPSS version 23.0 (SPSS, IBM Corp.).

## Ethics statement

The study was conducted in accordance with the guidelines of the Declaration of Helsinki and the principles of Good Clinical Practice. Ethics Committee approval was obtained from Research Ethics Board of each individual centers belonging to the GEPPO cohort. Ethics approval was obtained by competent Ethics Committees (protocol number 0004013, reference 135/2016, Clinica Universitaria di Malattie Infettive, P.O. Amedeo di Savoia, Torino, as coordinating center).

Because this was a retrospective and non-pharmacological study, informed consent has not been provided since in Italy ethical authorization for these studies is not needed (Italian Guidelines for classification and conduction of observational studies, established by the Italian Drug Agency, “Agenzia Italiana del Farmaco–AIFA” on March 20, 2008).

All data were fully anonymized before the statistical analysis was performed.

## Results

We included 210 women (17%) out of 1237 patients. Overall median age was 69.8 years old (67.1–73.9). No significant differences in HIV duration has been observed between males and females [16.3 (10.3–21.5) vs. 15.6 (10.1–20.8), p = 0.436 in females and males respectively] (Table 1).

**Table 1:** Characteristics of patients according to gender. Data are described as number (percentage) or median (interquartile range).

Variable	Females (n = 210)	Males (n = 1027)	Total (N = 1237)	P
**Age**—years	70.6 (67.3–74.8)	69.6 (67.1–73.8)	69.8 (67.1–73.9)	0.088
**Caucasian ethnicity**	203 (96.7%)	1013 (98.6%)	1216 (98.3%)	0.070
**HIV duration**—years	15.6 (10.1–20.8)	16.3 (10.4–21.5)	16.3 (10.3–21.3)	0.436
**current CD4+ cell/mm**^**3**^	613 (437–802)	619 (440–790)	618 (440–793)	0.606
**CD4+/CD8+ ratio**	0.97 (0.58–1.29)	0.78 (0.54–1.16)	0.81 (0.54–1.19)	**0.007**
**Nadir CD4+ cell/mm**^**3**^	189 (86–286)	198 (82–313)	196 (83–308)	0.484
**HIV RNA <50 copies/ml**	136 (74.3%)	740 (81.8%)	876 (80.5%)	**0.024**
**HCV Ab+**	21 (10%)	104 (10.2%)	125 (10.1%)	0.956
**HBsAg +**	14 (6.7%)	77 (7.5%)	91 (7.3%)	0.674

**Fig 1:**
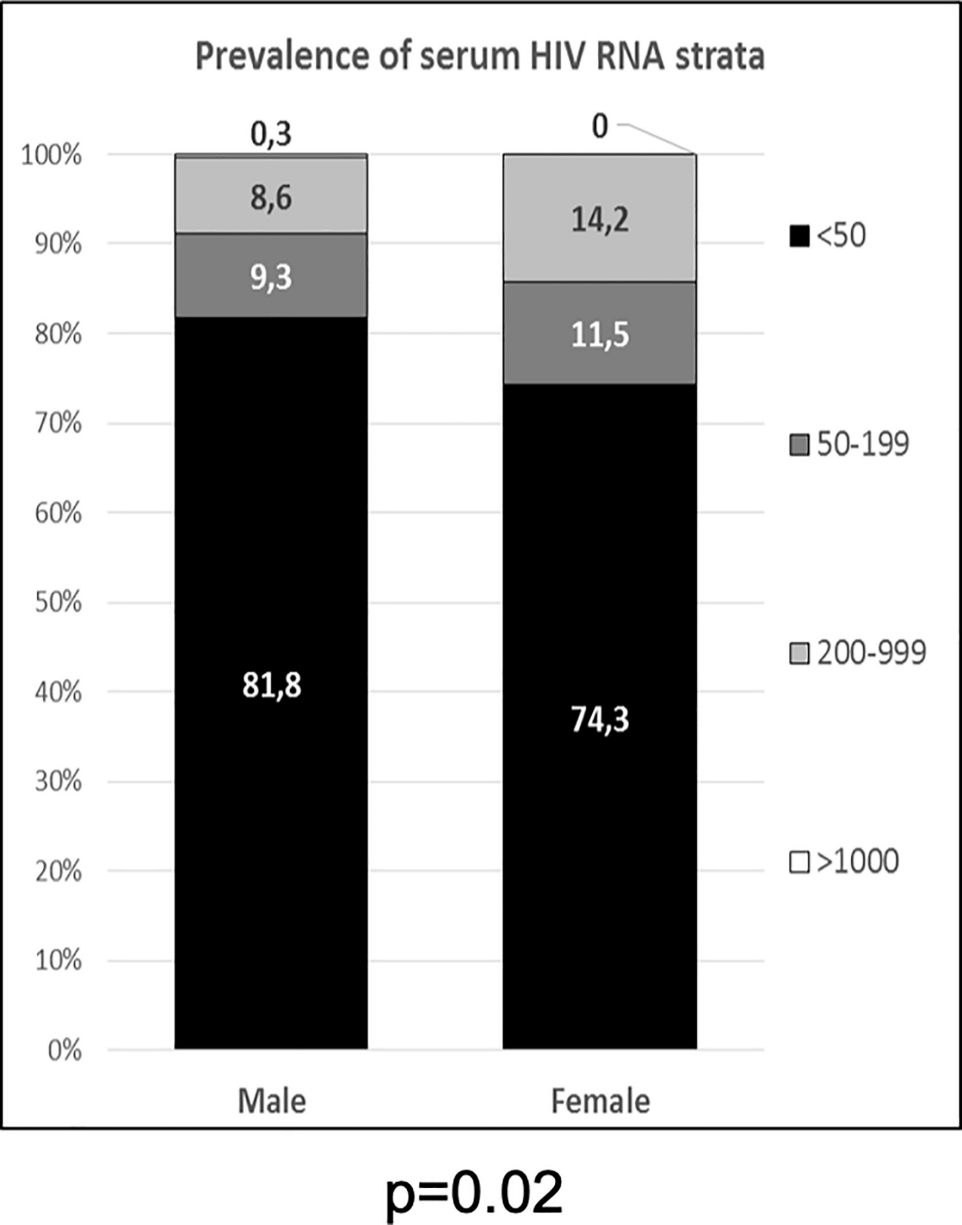
Current plasma HIV RNA. Current plasma HIV RNA according to gender and stratified by: < 50 copies/mL; between 50 and 199 copies/mL, between 200 and 999 copies/mL; >1000 copies/mL.

**Fig 2:**
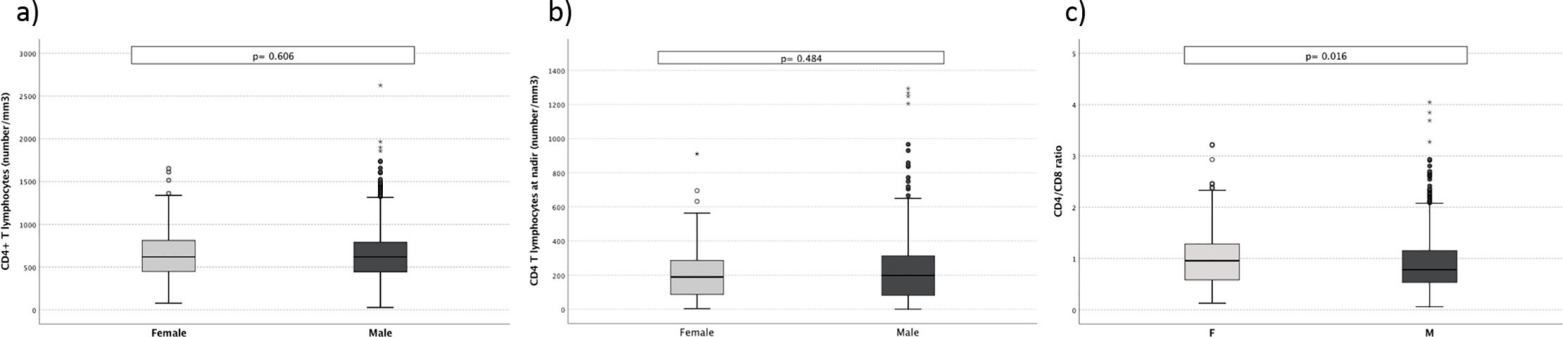
Immunological parameters according to gender. Immunological parameters between females and males according to current CD4+T lymphocytes (panel a); CD4+ T lymphocytes nadir (panel b); CD4+/CD8+ ratio (panel c).

Elderly HIV-positive women were less likely to present a HIV-RNA <50 copies/mL (74.3% vs. 81.8%; OR 0.64, 95%CI 0.44–0.93; p = 0.024) while we did not find any differences in terms of plasma HIV RNA levels between females and males with detectable (>50 copies/ml) viral load (p = 0.559) (Fig 1). Female participants showed higher CD4+/CD8+ ratio [0.97 (0.58–1.29) vs. 0.78 (0.53–1.16), p = 0.007] but similar current and nadir CD4+ T-cell counts. (Fig 2A, 2B and 2C). A nadir below 200 cells/mm^3^ was observed in 50.6% participants with no difference according to gender.

To check whether gender was independently associated the outcomes of interest we performed a binary logistic regression analysis (virological suppression) and a linear regression analysis (higher CD4/CD8 ratio) including age, current CD4+ T-cell count, nadir CD4+ t-cell count, type of treatment, multimorbidity and polypharmacy as covariates. We found that female gender was independently associated with a higher CD4/CD8 ratio (p = 0.003) but not with virological suppression.

Current antiretroviral regimens were similar between the two genders (p>0.05). Triple regimens were the most frequently used in both populations, being prescribed globally in about 70% of patients. Globally, women were less likely to be treated with protease inhibitors (PIs) (39.5% vs 46.8%, p = 0.05) (Table 2). In triple-drug regimens females received less PIs (28% vs 38%, p = 0.022) and more integrase inhibitors (30% vs. 20%, p = 0.012). In females with bone disease PIs and boosted regimens (38% vs. 53.7% p = 0.026 and 30.4 vs 44.0% p = 0.048, respectively) were prescribed less frequently compared to males with bone disease.

**Table 2:** Antiretroviral therapies, co-morbidities and polypharmacy according to gender.

Variable	Females (n = 210)	Males (n = 1027)	Total (n = 1237)	p
**ARV therapies**				
Mono-dual	60 (28.6%)	299 (29.1%)	359 (29%)	
Triple	147 (70%)	711 (69.2%)	858 (69.3%)	0.338
Other	3 (1.4%)	17 (0.3%)	20 (1.61%)	
**Regimen including**:				
PI	83 (39.5%)	481 (46.8%)	564 (45.5%)	0.05
NNRTI	86 (41%)	441 (42.9%)	527 (42.6%)	0.456
InSTI	75 (35.7%)	305 (29.7%)	380 (30.7%)	0.118
**NRTI-sparing regimens**	52 (24.8%)	278 (27.1%)	330(26.6%)	0.298
**TDF-sparing regimens**	157 (74.8%)	742 (72.2%)	899 (72.6%)	0.314
**Unboosted regimens**	143 (68.1%)	642 (62.5%)	785 (63.4%)	0.079
**Mean comorbidities** (±SD)	2.19 (±1.42)	2.37 (±1.42)	2.34 (±1.42)	0.070
**Comorbidities**				
CVD	14 (9.5%	154 (22.8%)	168 (20.46%)	**<0.001**
CKD	34 (21.3%)	150 (20.8%)	184 (20.8%)	0.381
Hypertension	113 (65.3%)	456 (64.5%)	569 (64.6%)	0.206
T2DM	38 (24.5%)	201 (29.1%)	239 (28.3%)	0.696
Bone disease	79 (48.8%)	134 (22.9%)	213 (28.5%)	**<0.001**
Hyperlipidemia	134 (75.3%)	497 (70.5%)	631 (71.4%)	0.076
COPD	7 (4.8%)	57 (8.6%)	64 (7.9%)	0.191
Cancer	30 (16%)	147 (22.3%)	177 (20.92%)	0.761
**Polypharmacy** (≥5 drug excluded cART)	42 (20%)	234 (22.8%)	254 (20.5%)	0.326

SD = standard deviation, ARV = antiretroviral, PI = Protease Inhibitors, NNRTI = Non-Nucleoside Reverse Transcriptase Inhibitors, InSTI = Integrase Strand Transfer Inhibitors, NRTI = Nucleos(t)ide Reverse Transcriptase Inhibitors, TDF = Tenofovir Disoproxil Fumarate, CVD = Cardiovascular Disease, CKD = Chronic Kidney Disease, COPD = Chronic Obstructive Pulmonary Disease, cART = combination antiretroviral therapy.

Hyperlipidemia, hypertension and bone disease (75.3, 65.3 and 48.8%, respectively) were the most frequent comorbidities in the female population. When the two populations were compared, bone disease was more common in females (48.8% vs 22.9%, p<0.001) while males presented more frequently cardiovascular disease (22.8% vs 9.5%, p<0.001).

Polypharmacy was not uncommon and similarly frequent in both genders, being present in 20% of cases. A higher use of lipid-lowering drugs (20.5% vs. 14.8%, p = 0.04) was observed in female participants. Anyway, elderly women were less likely to receive anti-thrombotic agents (18.6% vs. 26.3%, p = 0.019) even when cardiovascular disease was recorded (57.1% vs. 83.1%, p = 0.018). ACE inhibitors were less commonly used in female patients (27.1% vs. 33%, p = 0.09), especially in the presence of type 2 diabetes mellitus (2.6% vs. 13.9%, p = 0.006).

## Discussion

To our best knowledge, this is the first study including such large sample size of geriatric women living with HIV. Some cohort studies [12,13,25] included much lower numbers of HIV-positive women, where geriatric age was not always defined as ≥65 years old.

From a virologic point of view, our results showed that women presented a lower level of virologic suppression when compared to males (74.3% vs. 81.8%, p = 0.02).

This finding was unexpected, as long as lower viral loads has been described for women in comparison to men in several studies, in both naïve and cART-experienced patients [23,26,27, 28]. Gender-based differences in viral load may disappear along the progression of the infection [27]. Anyway, in a cohort of naïve patients with CD4+ T cells counts <300 cell/mmc from Grinsztejn *et al* [29], women showed lower values of HIVRNA also for more advanced stages of the disease. Therefore, we hypothesized that females may have a worse virologic control because of a lower adherence to cART as previously observed [23,30], or maybe a higher rate of intolerance to cART drugs or a possible presence of DDis [23]. Unfortunately, the study design did not allow to explore such factors. Moreover, as long as we tried to explore the existence of a correlation between virological replication in females and some variables such as type of cART, comorbidities and presence of polypharmacy, no statistical significance was found.

In our study, the nadir of CD4+ T-cell count was often <200 cell/mmc in both females and males, which is in line with the study from Tavoschi *et al* [31] showing that CD4+ T-cell count at diagnosis tends to be lower than 350 cell/mmc in older patients. Despite several studies highlighted increased rates of HIV diagnosis in people aged 50 years or more [32–33], elderly individuals are more likely to present late diagnosis of HIV infection [34–36]. A delay in HIV diagnosis can be especially threatening in this population because of higher rates of mortality, especially when an AIDS-defining event is present [37–39]. Moreover, in our analysis females did not show a significant lower nadir of CD4+ T-cells when compared to males (189 vs 198 cell/mm^3^, p = 0.48, respectively). However, as long as more than 50% of our patients showed a CD4+ T-cell count nadir <200 cell/mm^3^, we can hypothesize a lack of attention from both clinicians and prevention strategies in this population, which may perceive a lower risk of sexually transmitted diseases in this population. Certainly, a low self-perception of the risk and a lack of knowledge is present in older women about HIV infection and its transmission patterns.

Nevertheless, despite worse virologic outcomes, women displayed better CD4+/CD8+ ratio in comparison to men (1.01 vs 0.9, p = 0.016). This result was confirmed in multivariate analysis, where female gender was an independent predictor of higher CD4+/CD8+ ratio.

This is in line with a recent study, which has demonstrated that female gender is a strong predictor of immune reconstitution [40], where other studies observed the same trend in the past years [41].

With regard to antiretroviral regimens, no differences between the two genders were found in relation to the use of less-drug regimens. In both cases, standard triple regimens were prescribed in about two thirds of patients. Anyway, we observed a slight lower prescription of PIs in females, in both standard and less-drug regimens. In the study from Menzaghi *et al* [30], women were more likely to interrupt PIs. Specifically, women on atazanavir-based regimens were more likely to discontinue or switch treatment because of grade 1–2 adverse events, low adherence and patient’s will, when compared to men. Moreover, in our cohort, females with bone disease were less likely to be prescribed with PIs and boosted regimens. Pathology of osteoporosis in HIV-positive patients is complex and has been correlated to several factors, where its correlation with different class of drugs remained controversial [42–43]. In a previous study, we observed an overall increase of bone turnover in HIV-positive patients starting cART with tenofovir/emtricitabine plus either atazanavir/ritonavir or efavirenz, where markers of bone resorption were higher in the first group, compared to the latter [44]. On the other hand, in a study from Yin *et al*, which analyzed bone loss in HIV-positive post-menopausal women, the annualized rates of bone loss adjusted for baseline bone mass density (BMD) did not differ in women on PI-based in comparison to NNRTI-based ART at any site, while it was greater among those on treatment with tenofovir [45].

Whether our clinicians chose to prescribe less PIs in order to try to avoid metabolic effects and eventual drug-to-drug interactions in the female population, or whether this choice was secondary to patients adherence and/or adverse events remains uncertain, but could represent a future matter of research.

Analyzing co-morbidities, although the mean number of co-morbidities did not differ between the two genders, we found a significant higher prevalence of bone disease in females, while CVD was more prevalent in males. In HIV-positive patients, regardless of age and gender, bone disease is more frequent in comparison to the general population, in terms of either presence of osteopenia/osteoporosis and/or asymptomatic vertebral fracture [46,47].

As expected, we found a higher prevalence of CVD in males. However, among people living with HIV, CVD is frequently observed also in females and higher when compared to HIV-negative females [48]. It is important to consider that CVD is common among females overall but often underestimated due to the fact of the challenges in atypical clinical presentation and differential diagnosis.

Polypharmacy was present in one out of five patients, irrespectively of gender. Lipid-lowering drugs were more prescribed in females compared to men. These findings showed that clinicians involved in HIV care seem to pay attention to the overall risk related to hyperlipidemia in females living with HIV, although we did not perform a specific analysis on the appropriateness of prescriptions. Given that HIV *per se* and exposure to antiretroviral drugs have been related to deregulations in plasmatic lipids in both males and females [48], maybe the higher prescriptions of lipid-lowering drugs in the female population can be related to the awareness of clinicians about metabolic changes occurring after menopause.

As our study shows, clinicians seem not to pay the same attention for what concerns CVD in elderly females. In fact, despite previous data showed an increased risk of CV events in HIV-positive women [49], females were less likely to receive anti-thrombotic agents even though CVD had been present in the patient history [50,51]. In fact, there is a higher risk of relapse of CV event potentially related to DDIs between anti-thrombotic agents and antiretrovirals [52].

This study has some limitations: first, data of the sample of HIV-infected patients were collected within the routinary clinical practice, but they were retrospectively analyzed. Therefore, some data that could have been interesting to show and report, such as previous cART regimes, the existence of any previous virological failures, duration of cART, pre-cART CD4+/CD8+ ratios, were not collected. Secondly, only seven major co-morbidities were explored, thus not allowing to draw a fully comprehensive profile of the patients we studied. Moreover, because of the cross-sectional nature of our study is that we couldn’t study whether the higher viremic loads in women were due to viral load blips or to virological failure.

## Conclusions

This study shows that a better knowledge of this special population is necessary for the years to come. Where some differences are already present in the management of elderly HIV-positive women in comparison to men, some other may be further investigated and addressed. Especially, we believe that prevention campaigns should start to consider this population, which is actually forgotten, in order to firstly avoid HIV-infection and secondly, to diagnose it at earlier stages. Moreover, a better understanding of the characteristics and of the specific needs of these population may help for a better management and retention in care for the near, and farer, future. A gender-medicine approach should be warranted in all settings.

## Supporting information

S1 Data Anon. (XLSX)
